# Supplementary material for: The MOBH35 Metal–Organic Barrier Heights Reconsidered: Performance of Local-Orbital Coupled Cluster Approaches in Different Static Correlation Regimes
Source: J Chem Theory Comput. 2022 Jan 19;18(2):883–98. doi: 10.1021/acs.jctc.1c01126 (PMC8830049; doi:10.1021/acs.jctc.1c01126)
Supplement: Supplementary file 1 — ct1c01126_si_001.pdf [file ct1c01126_si_001.pdf]

Supporting Information

The MOBH35 metal-organic barrier heights reconsidered: performance of local-orbital coupled cluster approaches in different static correlation regimes

Emmanouil Semidalas [1] *and Jan M.L. Martin*[1, a)]

[1] Department of Molecular Chemistry and Materials Science, Weizmann Institute of Science, 7610001 Reḥovot, Israel

Email: gershom@weizmann.ac.il

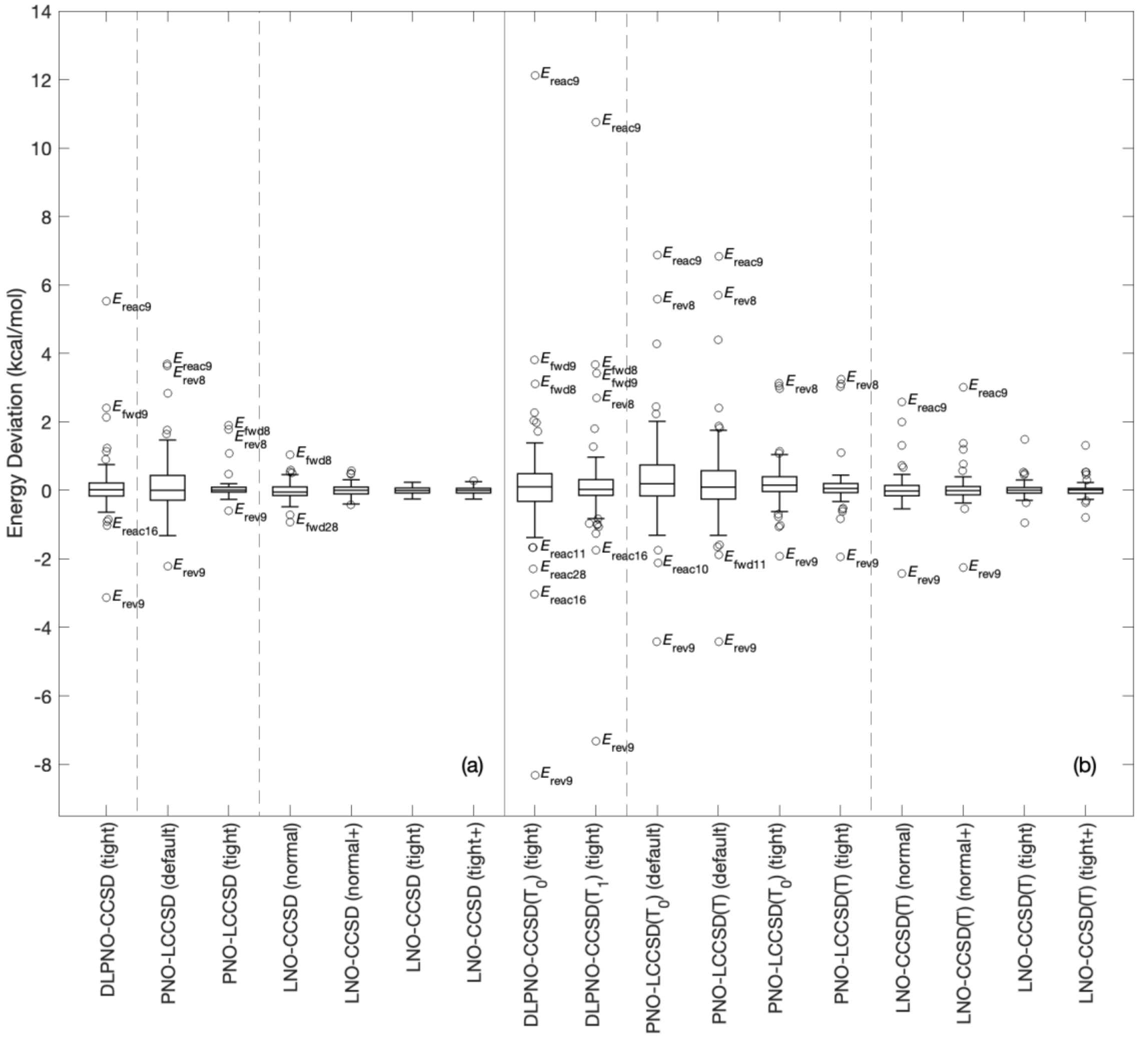

Figure S1. Box-and-whisker plot for the energy deviations of local CC methods (the precision criteria are indicated in parentheses; for LNO-CCSD(T): Normal (wpairtol = 1e-5), Normal+ (wpairtol = 1e-6), Tight (wpairtol = 3e-6), Tight+ (wpairtol = 1e-6)) from (a) canonical CCSD; (b) canonical CCSD(T). The def2-SV(P) basis set was used throughout.

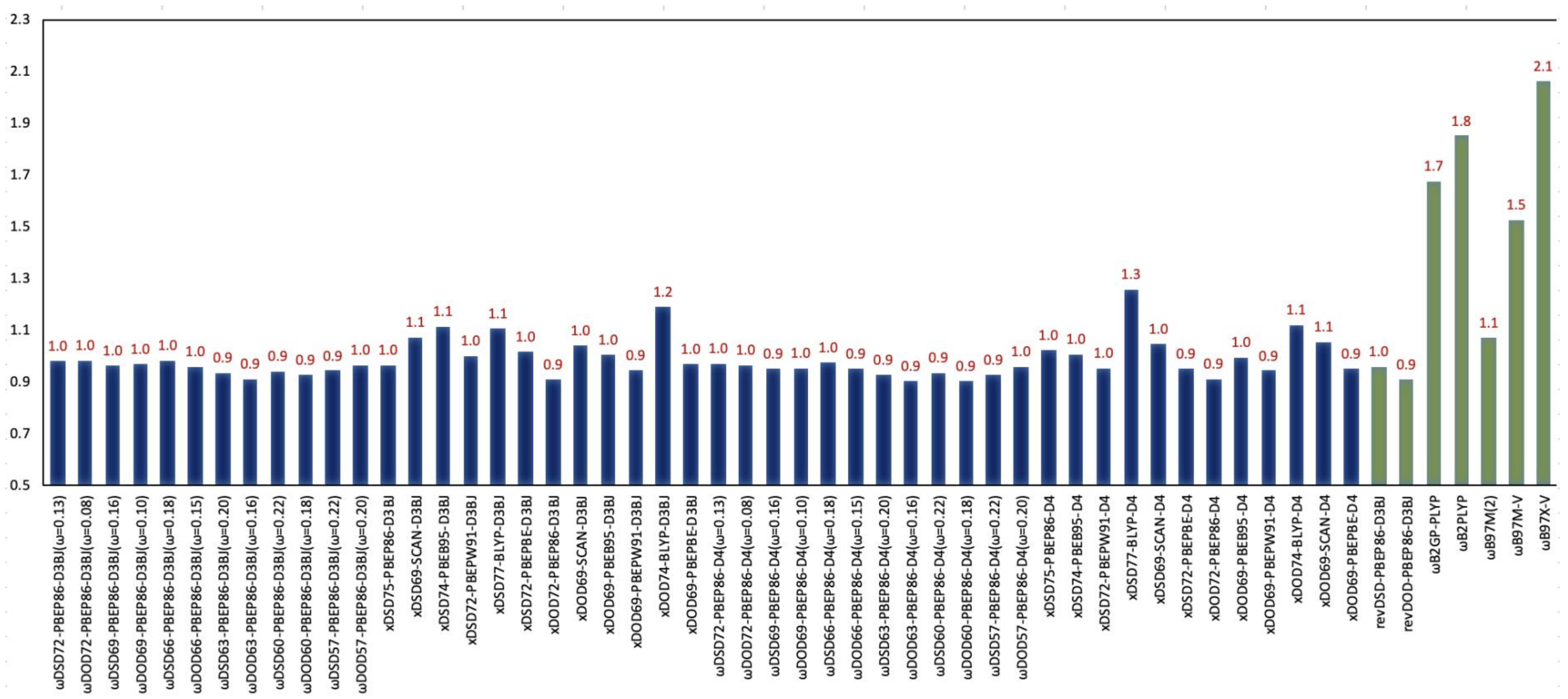

Figure S2. MAD (kcal/mol) statistics for xDSD and ωDSD functionals evaluated against the modified MOBH35 dataset and the revised reference energies reported in this work. Reactions 9, 11, 12, and 17-20 were not included in this comparison.

Table S1. Data for boxplot with quartiles and median from LNO-CCSD(T, Tight+)/def2-TZVPP + $\Delta E_{[CCSD(T)-LNO-CCSD(T)]/def2-SVP}$ reference values.

Ref.			def2-TZVPP LNO-CCSD(T) Tight+wpt **LNO Tight+wpt**	def2-TZVPP LNO-CCSD(T) Tight **LNO Tight**	def2-TZVPP LNO-CCSD(T) Normal **LNO Normal**	def2-TZVPP LNO-CCSD(T) Normal+ **LNO Normal+**	def2-TZVPP DLPNO-CCSD(T1) Tight T1 **DLPNO-T1 Tight**	def2-TZVPP DLPNO-CCSD(T) Tight T0 **DLPNO-T0 Tight**	def2-TZVPP PNO-LCCSD(T) Tight T **PNO Tight T**	def2-TZVPP PNO-LCCSD(T0) Tight T0 **PNO Tight T0**	def2-TZVPP PNO-LCCSD(T) Normal T **PNO Normal T**	def2-TZVPP PNO-LCCSD(T0) Normal T0 **PNO Normal T0**
26.76	**01_end**	ΔE#fwd	-0.02	-0.01	0.00	0.05	0.08	0.37	0.02	0.28	-0.38	-0.12
13.94	**01_start**	ΔE#rev	-0.03	-0.04	-0.05	-0.11	0.20	0.59	-0.16	0.14	-0.54	-0.24
12.82	**01_ts**	ΔE_R	0.02	0.03	0.05	0.15	-0.12	-0.22	0.18	0.13	0.16	0.12
5.92	**02_end**	ΔE#fwd	-0.02	0.00	0.14	0.09	-0.06	-0.11	0.13	0.21	0.07	0.15
22.38	**02_start**	ΔE#rev	-0.10	-0.09	-0.14	-0.11	-0.08	0.14	-0.20	-0.14	-0.46	-0.40
-16.46	**02_ts**	ΔE_R	0.08	0.09	0.27	0.20	0.03	-0.25	0.33	0.35	0.53	0.55
0.99	**03_end**	ΔE#fwd	-0.01	-0.01	-0.01	-0.02	-0.01	0.06	0.07	0.15	0.04	0.12
26.29	**03_start**	ΔE#rev	-0.24	-0.24	-0.52	-0.52	-0.92	-0.02	0.18	0.64	0.12	0.59
-25.31	**03_ts**	ΔE_R	0.23	0.23	0.51	0.50	0.91	0.08	-0.11	-0.49	-0.07	-0.46
1.53	**04_end**	ΔE#fwd	0.02	0.02	-0.01	-0.01	0.13	0.00	-0.12	-0.15	-0.16	-0.18
7.81	**04_start**	ΔE#rev	-0.05	-0.05	-0.17	-0.18	-0.24	0.62	0.19	0.61	0.18	0.60
-6.28	**04_ts**	ΔE_R	0.07	0.07	0.16	0.17	0.37	-0.62	-0.32	-0.75	-0.34	-0.79
4.75	**05_end**	ΔE#fwd	-0.06	-0.06	-0.43	-0.29	-0.57	-0.51	-0.18	-0.10	-0.49	-0.42
22.73	**05_start**	ΔE#rev	-0.09	-0.08	-0.13	-0.14	0.18	-0.18	-0.15	0.03	-0.05	0.12
-17.98	**05_ts**	ΔE_R	0.03	0.03	-0.30	-0.16	-0.75	-0.33	-0.03	-0.13	-0.44	-0.54
15.60	**06_end**	ΔE#fwd	-0.14	-0.13	-0.25	-0.20	-0.26	-0.15	0.14	0.38	0.13	0.37
14.76	**06_start**	ΔE#rev	-0.20	-0.17	-0.37	-0.28	-0.66	-0.72	0.15	0.23	-0.13	-0.05
0.84	**06_ts**	ΔE_R	0.06	0.04	0.12	0.08	0.40	0.58	-0.01	0.14	0.27	0.42
27.66	**07_end**	ΔE#fwd	-0.10	-0.08	-0.02	0.05	0.29	0.59	0.17	0.40	0.31	0.53
18.97	**07_start**	ΔE#rev	-0.10	-0.11	-0.12	-0.06	-0.55	-0.30	-0.18	0.08	0.03	0.27
8.69	**07_ts**	ΔE_R	0.00	0.04	0.10	0.11	0.84	0.89	0.36	0.33	0.29	0.26
34.69	**08_end**	ΔE#fwd	0.52		1.60	1.42	3.68	3.01	3.52	3.36	4.54	4.39
31.48	**08_start**	ΔE#rev	0.19	0.19	0.74	1.18	3.26	2.47	3.67	3.50	6.32	6.13
3.21	**08_ts**	ΔE_R	0.33		0.86	0.24	0.42	0.54	-0.15	-0.14	-1.78	-1.74
27.83	**09_end**	ΔE#fwd	0.49	0.49	0.22	0.74	3.70	4.43	1.50	1.54	2.94	2.98
12.67	**09_start**	ΔE#rev	-1.05	-1.05	-1.98	-1.95	-7.41	-8.23	-1.87	-1.82	-3.82	-3.80

15.16	**09_ts**	ΔE_R	1.54	1.54	2.19	2.69	11.10	12.66	3.36	3.36	6.76	6.78
-3.79	**10_end**	ΔE#fwd	-0.18	-0.17	-0.43	-0.44	-0.87	-0.88	-0.52	-0.33	-1.00	-0.80
9.02	**10_start**	ΔE#rev	0.22	0.26	0.30	0.14	0.33	0.51	0.30	0.78	0.80	1.27
-12.81	**10_ts**	ΔE_R	-0.40	-0.43	-0.73	-0.58	-1.20	-1.39	-0.82	-1.11	-1.79	-2.08
29.84	**11_end**	ΔE#fwd	-0.01	0.00	0.02	-0.04	-0.60	-0.53	-0.43	0.01	-0.95	-0.50
82.91	**11_start**	ΔE#rev	0.00	0.01	0.61	0.67	0.80	1.33	0.44	1.61	0.25	1.38
-53.07	**11_ts**	ΔE_R	-0.01	-0.01	-0.59	-0.71	-1.40	-1.86	-0.87	-1.60	-1.20	-1.89
5.48	**12_end**	ΔE#fwd	-0.01	-0.01	-0.22	-0.08	-0.11	-0.13	-0.06	-0.17	-0.06	-0.16
37.23	**12_start**	ΔE#rev	0.14	0.16	0.27	0.39	-0.07	-0.06	-0.07	-0.20	0.14	0.01
-31.74	**12_ts**	ΔE_R	-0.15	-0.17	-0.49	-0.47	-0.04	-0.07	0.01	0.03	-0.20	-0.17
20.64	**13_end**	ΔE#fwd	-0.07	-0.10	-0.02	0.07	1.68	2.19	0.37	0.84	1.33	1.79
48.48	**13_start**	ΔE#rev	0.14	0.10	0.25	0.33	1.27	2.20	0.49	0.80	1.18	1.48
-27.85	**13_ts**	ΔE_R	-0.21	-0.19	-0.26	-0.26	0.41	-0.01	-0.12	0.04	0.15	0.31
10.24	**14_end**	ΔE#fwd	-0.01	-0.01	0.05	0.08	0.21	0.39	0.10	0.26	0.15	0.30
14.44	**14_start**	ΔE#rev	-0.06	-0.05	-0.03	-0.02	-0.16	-0.22	-0.03	0.15	-0.32	-0.14
-4.21	**14_ts**	ΔE_R	0.05	0.05	0.08	0.10	0.36	0.61	0.13	0.10	0.46	0.44
20.79	**15_end**	ΔE#fwd	0.03	0.04	0.04	0.04	-0.07	0.31	0.00	0.26	0.45	0.70
74.68	**15_start**	ΔE#rev	0.03	0.04	-0.02	-0.04	0.86	2.22	0.75	1.61	1.71	2.56
-53.89	**15_ts**	ΔE_R	0.00	0.00	0.06	0.08	-0.92	-1.92	-0.75	-1.35	-1.26	-1.86
35.51	**16_end**	ΔE#fwd	-0.02	-0.02	-0.19	-0.18	-1.40	-2.17	-0.77	-0.67	-1.29	-1.20
53.51	**16_start**	ΔE#rev	0.08	0.08	0.18	0.12	1.29	1.78	0.32	0.59	0.56	0.83
-18.00	**16_ts**	ΔE_R	-0.10	-0.10	-0.37	-0.30	-2.70	-3.95	-1.08	-1.26	-1.85	-2.02
8.09	**21_end**	ΔE#fwd	0.10	0.08	0.32	0.35	0.32	0.47	-0.09	-0.02	-0.33	-0.26
8.10	**21_start**	ΔE#rev	0.09	0.08	0.32	0.34	0.32	0.48	-0.10	-0.02	-0.34	-0.26
-0.01	**21_ts**	ΔE_R	0.00	0.00	0.01	0.00	0.00	0.00	0.00	0.00	0.01	0.01
14.27	**22_end**	ΔE#fwd	0.18	0.16	0.28	0.32	0.58	0.68	0.43	0.70	0.59	0.85
27.01	**22_start**	ΔE#rev	0.33	0.31	0.53	0.58	0.94	1.40	0.48	0.87	0.99	1.37
-12.73	**22_ts**	ΔE_R	-0.15	-0.16	-0.25	-0.26	-0.36	-0.73	-0.05	-0.17	-0.40	-0.52
29.97	**23_end**	ΔE#fwd	-0.01	N/A	0.26	0.33	0.81	1.36	0.72	1.04	0.63	0.92
20.40	**23_start**	ΔE#rev	-0.07	N/A	-0.14	-0.04	0.53	1.40	0.47	0.95	1.03	1.47
9.57	**23_ts**	ΔE_R	0.05	0.06	0.41	0.37	0.28	-0.05	0.25	0.09	-0.40	-0.55

25.42	**26_end**	ΔE#fwd	-0.09	-0.09	-0.10	-0.11	0.22	0.26	0.01	0.10	0.05	0.14
0.08	**26_start**	ΔE#rev	0.00	0.01	0.01	0.00	-0.04	-0.01	0.03	0.03	0.01	0.00
25.34	**26_ts**	ΔE_R	-0.10	-0.09	-0.11	-0.11	0.26	0.27	-0.02	0.07	0.05	0.14
14.03	**27_end**	ΔE#fwd	0.03	0.04	-0.08	-0.07	0.01	-0.29	0.03	-0.02	0.13	0.08
2.01	**27_start**	ΔE#rev	-0.08	-0.08	-0.14	-0.11	-0.06	-0.18	-0.13	-0.05	0.09	0.17
12.02	**27_ts**	ΔE_R	0.12	0.12	0.06	0.05	0.08	-0.11	0.16	0.03	0.04	-0.09
30.47	**28_end**	ΔE#fwd	0.18	0.18	0.12	0.20	-0.13	-1.25	0.10	0.38	0.00	0.28
15.87	**28_start**	ΔE#rev	-0.01	-0.01	-0.13	-0.04	0.46	1.17	0.18	0.47	0.25	0.53
14.60	**28_ts**	ΔE_R	0.19	0.18	0.25	0.24	-0.59	-2.42	-0.08	-0.09	-0.24	-0.25
15.10	**29_end**	ΔE#fwd	0.16	0.17	0.08	0.17	0.31	0.53	0.28	0.41	0.68	0.80
31.94	**29_start**	ΔE#rev	0.06	0.03	-0.29	-0.16	0.07	0.07	0.23	0.47	0.56	0.79
-16.84	**29_ts**	ΔE_R	0.10	0.13	0.37	0.33	0.24	0.46	0.05	-0.06	0.12	0.01
10.04	**30_end**	ΔE#fwd	0.10	0.11	0.09	0.14	0.02	0.05	0.11	0.28	0.08	0.25
16.92	**30_start**	ΔE#rev	-0.03	-0.03	-0.03	-0.03	-0.02	0.24	0.06	0.10	-0.35	-0.31
-6.88	**30_ts**	ΔE_R	0.13	0.14	0.11	0.18	0.04	-0.19	0.05	0.18	0.44	0.56
3.24	**31_end**	ΔE#fwd	0.07	0.04	-0.24	0.00	0.26	0.13	0.33	0.69	1.49	1.84
13.32	**31_start**	ΔE#rev	0.05	0.06	0.01	-0.05	0.09	0.56	0.43	0.55	1.25	1.36
-10.08	**31_ts**	ΔE_R	0.02	-0.02	-0.26	0.05	0.17	-0.43	-0.10	0.14	0.24	0.48
20.58	**32_end**	ΔE#fwd	-0.08	-0.08	-0.14	-0.12	-0.75	-0.87	-0.07	0.86	-0.19	0.72
61.82	**32_start**	ΔE#rev	0.18	0.18	0.24	0.20	0.72	0.74	0.38	1.42	0.67	1.69
-41.24	**32_ts**	ΔE_R	-0.26	-0.26	-0.38	-0.32	-1.48	-1.62	-0.45	-0.56	-0.86	-0.97
1.24	**33_end**	ΔE#fwd	-0.03	-0.03	-0.11	-0.11	-0.05	0.28	0.04	0.42	-0.02	0.35
8.19	**33_start**	ΔE#rev	-0.07	-0.07	-0.08	-0.08	-0.24	0.01	0.02	0.52	0.00	0.49
-6.95	**33_ts**	ΔE_R	0.04	0.04	-0.03	-0.03	0.20	0.27	0.03	-0.10	-0.01	-0.14
29.76	**34_end**	ΔE#fwd	-0.03	-0.02	0.01	-0.07	0.02	0.51	0.46	0.55	0.96	1.05
3.42	**34_start**	ΔE#rev	0.01	0.01	0.02	0.06	0.02	0.29	0.23	0.41	0.49	0.67
26.33	**34_ts**	ΔE_R	-0.04	-0.03	-0.01	-0.13	0.00	0.23	0.22	0.14	0.47	0.38
16.74	**35_end**	ΔE#fwd	0.00	0.00	0.20	0.23	0.63	0.91	0.17	0.42	0.53	0.77
-2.58	**35_start**	ΔE#rev	0.08	0.10	0.19	0.07	0.34	0.42	0.30	0.18	0.87	0.74
19.32	**35_ts**	ΔE_R	-0.08	-0.10	0.01	0.17	0.29	0.49	-0.13	0.24	-0.34	0.02

Table S1. Data for boxplot with quartiles and median from LNO-CCSD(T, Tight+)/def2-TZVPP + $\Delta E_{[CCSD(T)-LNO-CCSD(T)]/def2-SVP}$ reference values (continued)

		LNO Tight+wpt	LNO Tight	LNO Normal	LNO Normal+	DLPNO-T1 Tight	DLPNO-T0 Tight	PNO Tight T	PNO Tight T0	PNO Normal T	PNO Normal T0
	MAX	1.54	1.54	2.19	2.69	11.10	12.66	3.67	3.50	6.76	6.78
	97.5%	0.47	0.31	0.84	1.11	3.62	2.93	3.08	3.10	4.30	4.18
	%90	0.19	0.18	0.34	0.36	0.88	1.40	0.46	0.90	1.09	1.47
box top	75%	0.08	0.08	0.18	0.17	0.37	0.57	0.27	0.50	0.51	0.73
median	50%	0.00	0.00	0.01	0.00	0.08	0.13	0.05	0.15	0.08	0.25
X	AVG	0.02	0.02	0.03	0.06	0.19	0.22	0.15	0.28	0.24	0.36
box bottom	25%	-0.07	-0.07	-0.14	-0.11	-0.12	-0.22	-0.10	-0.04	-0.33	-0.21
	10%	-0.14	-0.15	-0.37	-0.29	-0.80	-1.03	-0.36	-0.39	-0.90	-0.79
	2.5%	-0.26	-0.26	-0.58	-0.57	-1.47	-2.38	-0.86	-1.34	-1.79	-2.00
	MIN	-1.05	-1.05	-1.98	-1.95	-7.41	-8.23	-1.87	-1.82	-3.82	-3.80
**RMSD**		**0.25**	**0.24**	**0.45**	**0.48**	**1.70**	**1.98**	**0.77**	**0.88**	**1.39**	**1.47**
**IQR (interquartile range)**		**0.15**	**0.16**	**0.33**	**0.29**	**0.49**	**0.79**	**0.36**	**0.53**	**0.84**	**0.94**
top whisker		0.31	0.31	0.67	0.60	1.10	1.75	0.81	1.29	1.76	2.14
bottom whisker		-0.29	-0.31	-0.63	-0.54	-0.86	-1.40	-0.64	-0.83	-1.58	-1.63
top extreme fence		0.53	0.55	1.16	1.03	1.84	2.93	1.36	2.09	3.02	3.56
bottom extreme fence		-0.52	-0.54	-1.12	-0.97	-1.60	-2.59	-1.19	-1.63	-2.83	-3.04

Table S2. Data for boxplot with quartiles and median from CCSD(T)/def2-SVP reference values.

Ref.			def2-SVP LNO-CCSD(T) VeryTight **LNO VeryTight**	def2-SVP LNO-CCSD(T) Tight+wpt **LNO Tight+wpt**	def2-SVP LNO-CCSD(T) Tight **LNO Tight**	def2-SVP LNO-CCSD(T) Normal **LNO Normal**	def2-SVP DLPNO-CCSD(T1) Tight T1 **DLPNO-T1 Tight**	def2-SVP DLPNO-CCSD(T) Tight T0 **DLPNO-T0 Tight**	def2-SVP DLPNO-CCSD(T1) Normal T1 **DLPNO-T1 Normal**	def2-SVP PNO-LCCSD(T) Tight T1 **PNO Tight**	def2-SVP PNO-LCCSD(T0) Tight T0 **PNO-T0 Tight**	def2-SVP PNO-LCCSD(T) Normal T1 **PNO Normal**	def2-SVP PNO-LCCSD(T0) Normal T0 **PNO-T0 Normal**
27.06	**01_end**	ΔE#fwd	-0.02	-0.01	-0.01	-0.11	0.12	0.39	0.03	0.12	0.36	-0.19	0.05
14.02	**01_start**	ΔE#rev	0.01	-0.02	-0.03	-0.16	0.24	0.62	0.62	0.05	0.33	-0.55	-0.26
13.04	**01_ts**	ΔE_R	-0.04	0.02	0.02	0.05	-0.12	-0.23	-0.58	0.07	0.02	0.36	0.32
5.63	**02_end**	ΔE#fwd	-0.01	-0.03	-0.02	0.06	-0.05	-0.09	0.13	0.09	0.17	0.04	0.12
25.10	**02_start**	ΔE#rev	0.00	-0.11	-0.10	-0.14	0.05	0.25	0.11	0.04	0.12	-0.38	-0.30
-19.47	**02_ts**	ΔE_R	-0.01	0.08	0.08	0.20	-0.09	-0.34	0.01	0.05	0.05	0.42	0.42
0.95	**03_end**	ΔE#fwd	0.01	0.00	0.00	0.02	0.01	0.08	0.06	0.08	0.15	0.08	0.15
27.07	**03_start**	ΔE#rev	-0.11	-0.23	-0.24	-0.43	-0.55	0.38	-1.44	0.34	0.86	0.35	0.88
-26.12	**03_ts**	ΔE_R	0.12	0.23	0.23	0.45	0.56	-0.30	1.50	-0.26	-0.71	-0.27	-0.73
2.36	**04_end**	ΔE#fwd	0.02	0.02	0.02	-0.01	-0.07	-0.20	0.23	-0.15	-0.17	-0.14	-0.17
8.60	**04_start**	ΔE#rev	-0.06	-0.05	-0.05	0.01	-0.24	0.70	-0.59	0.13	0.62	0.19	0.68
-6.24	**04_ts**	ΔE_R	0.08	0.06	0.06	-0.02	0.17	-0.90	0.82	-0.28	-0.79	-0.33	-0.84
4.68	**05_end**	ΔE#fwd	-0.04	-0.04	-0.06	-0.29	-0.67	-0.68	-0.92	0.00	0.03	-0.56	-0.52
22.02	**05_start**	ΔE#rev	-0.03	-0.06	-0.07	-0.16	0.14	-0.21	0.22	0.18	0.30	0.31	0.43
-17.35	**05_ts**	ΔE_R	-0.02	0.02	0.01	-0.14	-0.81	-0.47	-1.14	-0.18	-0.27	-0.87	-0.95
13.44	**06_end**	ΔE#fwd	-0.09	-0.12	-0.14	0.05	0.10	0.22	0.59	0.18	0.37	0.30	0.48
13.56	**06_start**	ΔE#rev	-0.12	-0.21	-0.19	-0.28	-0.62	-0.74	-0.59	-0.02	0.02	0.05	0.08
-0.12	**06_ts**	ΔE_R	0.04	0.09	0.05	0.33	0.72	0.97	1.19	0.20	0.36	0.25	0.41
26.66	**07_end**	ΔE#fwd	-0.10	-0.11	-0.09	-0.08	0.09	0.30	-0.14	0.08	0.30	0.92	1.11
18.30	**07_start**	ΔE#rev	-0.10	-0.10	-0.11	-0.25	-0.88	-0.73	-0.48	-0.09	0.13	0.42	0.62
8.36	**07_ts**	ΔE_R	0.00	-0.01	0.02	0.17	0.97	1.03	0.34	0.17	0.17	0.50	0.49
36.92	**08_end**	ΔE#fwd	0.26	0.52	0.53	2.01	3.94	3.28	4.17	3.23	3.10	4.63	4.50
32.30	**08_start**	ΔE#rev	-0.05	0.18	0.19	1.09	2.97	2.19	3.51	3.27	3.14	5.74	5.59
4.62	**08_ts**	ΔE_R	0.31	0.34	0.34	0.92	0.97	1.09	0.66	-0.04	-0.04	-1.11	-1.09
28.59	**09_end**	ΔE#fwd	0.24	0.50	0.49	0.19	3.59	4.09	4.67	1.21	1.26	2.80	2.84

15.20	**09_start**	ΔE#rev	-0.33	-0.94	-1.06	-2.23	-7.50	-8.55	-10.34	-2.01	-1.99	-4.30	-4.30
13.39	**09_ts**	ΔE_R	0.57	1.44	1.55	2.42	11.09	12.63	15.02	3.23	3.25	7.10	7.14
-3.48	**10_end**	ΔE#fwd	-0.14	-0.20	-0.18	-0.06	-0.83	-0.87	-1.01	-0.55	-0.43	-1.08	-0.95
9.58	**10_start**	ΔE#rev	0.17	0.19	0.22	0.20	0.18	0.36	1.05	-0.18	0.43	0.37	0.97
-13.06	**10_ts**	ΔE_R	-0.31	-0.39	-0.40	-0.26	-1.01	-1.23	-2.06	-0.37	-0.86	-1.45	-1.91
29.81	**11_end**	ΔE#fwd	-0.10	-0.02	-0.01	0.04	-0.38	-0.28	-0.23	-0.62	-0.03	-1.68	-1.10
84.09	**11_start**	ΔE#rev	-0.05	-0.02	0.00	0.39	0.60	1.13	2.69	0.18	1.03	-0.28	0.53
-54.27	**11_ts.**	ΔE_R	-0.05	0.00	-0.01	-0.35	-0.98	-1.41	-2.92	-0.81	-1.07	-1.41	-1.64
5.67	**12_end**	ΔE#fwd	0.01	0.00	-0.01	-0.18	-0.18	-0.20	-0.30	-0.05	-0.16	0.09	-0.02
36.83	**12_start**	ΔE#rev	-0.04	0.16	0.14	-0.04	-0.18	-0.23	-1.01	-0.09	-0.19	0.09	-0.01
-31.16	**12_ts**	ΔE_R	0.05	-0.16	-0.15	-0.14	0.00	0.03	0.70	0.04	0.04	0.01	0.00
18.37	**13_end**	ΔE#fwd	0.00	-0.08	-0.07	-0.01	1.80	2.34	2.69	0.39	0.85	1.83	2.26
48.18	**13_start**	ΔE#rev	0.07	0.13	0.14	0.11	1.29	2.08	1.34	0.47	0.78	1.15	1.44
-29.81	**13_ts**	ΔE_R	-0.06	-0.21	-0.21	-0.12	0.51	0.25	1.35	-0.08	0.07	0.68	0.82
10.17	**14_end**	ΔE#fwd	-0.07	0.00	-0.01	0.00	0.15	0.32	0.37	0.12	0.23	0.28	0.38
13.38	**14_start**	ΔE#rev	-0.06	-0.06	-0.06	-0.13	-0.16	-0.21	0.23	-0.03	0.12	-0.22	-0.08
-3.21	**14_ts**	ΔE_R	-0.01	0.05	0.05	0.12	0.30	0.53	0.14	0.15	0.11	0.50	0.46
23.90	**15_end**	ΔE#fwd	0.00	0.03	0.03	0.06	0.11	0.43	0.25	0.10	0.37	0.68	0.94
74.84	**15_start**	ΔE#rev	0.00	0.03	0.03	-0.02	0.45	1.85	1.55	0.33	1.09	1.29	2.02
-50.94	**15_ts**	ΔE_R	0.00	0.00	0.00	0.08	-0.34	-1.42	-1.30	-0.23	-0.72	-0.60	-1.08
37.45	**16_end**	ΔE#fwd	-0.02	-0.01	-0.02	0.01	-0.76	-1.71	-1.96	-0.34	-0.33	-0.87	-0.85
55.56	**16_start**	ΔE#rev	0.01	0.07	0.08	0.15	1.03	1.53	1.88	0.12	0.38	0.20	0.45
-18.11	**16_ts**	ΔE_R	-0.03	-0.08	-0.10	-0.14	-1.79	-3.24	-3.83	-0.46	-0.70	-1.08	-1.30
11.11	**21_end**	ΔE#fwd	0.01	0.10	0.10	0.19	0.48	0.67	0.56	-0.06	0.09	-0.34	-0.20
11.11	**21_start**	ΔE#rev	0.00	0.09	0.09	0.19	0.44	0.63	0.46	-0.06	0.09	-0.34	-0.20
0.00	**21_ts**	ΔE_R	0.00	0.00	0.00	0.00	0.04	0.04	0.10	0.00	0.00	0.00	0.00
14.86	**22_end**	ΔE#fwd	0.00	0.20	0.18	0.31	0.04	0.15	0.34	0.23	0.48	0.96	1.18
30.88	**22_start**	ΔE#rev	0.13	0.35	0.33	0.47	0.69	1.14	0.75	0.36	0.65	1.51	1.78
-16.02	**22_ts**	ΔE_R	-0.13	-0.15	-0.15	-0.16	-0.64	-0.99	-0.41	-0.12	-0.17	-0.55	-0.60
29.48	**23_end**	ΔE#fwd	-0.07	-0.01	-0.01	0.17	0.70	1.14	2.62	0.73	1.01	0.78	1.03
20.80	**23_start**	ΔE#rev	-0.06	-0.06	-0.07	-0.22	0.43	1.24	0.98	0.29	0.74	0.92	1.35

8.68	**23_ts**	ΔE_R	-0.02	0.05	0.05	0.40	0.26	-0.10	1.64	0.45	0.27	-0.15	-0.31
21.92	**26_end**	ΔE#fwd	0.02	-0.11	-0.09	0.04	0.28	0.38	0.60	0.07	0.14	0.12	0.19
-0.07	**26_start**	ΔE#rev	-0.01	0.00	0.00	0.07	0.00	-0.01	-0.03	0.03	0.03	-0.03	-0.03
21.99	**26_ts**	ΔE_R	0.03	-0.10	-0.10	-0.03	0.28	0.38	0.64	0.03	0.11	0.15	0.22
16.09	**27_end**	ΔE#fwd	0.01	0.04	0.03	0.06	0.06	-0.18	0.09	0.11	0.09	0.17	0.15
1.29	**27_start**	ΔE#rev	-0.05	-0.07	-0.08	-0.08	0.05	0.00	-0.32	-0.05	0.00	0.31	0.37
14.81	**27_ts**	ΔE_R	0.06	0.11	0.12	0.14	0.01	-0.18	0.40	0.17	0.08	-0.14	-0.22
31.96	**28_end**	ΔE#fwd	0.06	0.18	0.18	0.21	-0.32	-0.98	-1.29	0.25	0.48	0.11	0.34
16.85	**28_start**	ΔE#rev	-0.02	-0.01	-0.01	-0.02	0.51	1.08	0.95	0.19	0.40	0.23	0.43
15.11	**28_ts**	ΔE_R	0.08	0.19	0.19	0.23	-0.83	-2.06	-2.24	0.06	0.08	-0.12	-0.09
15.74	**29_end**	ΔE#fwd	0.04	0.18	0.16	-0.11	0.21	0.38	0.42	0.24	0.31	0.51	0.57
33.86	**29_start**	ΔE#rev	0.02	0.10	0.06	-0.10	-0.13	-0.16	0.01	0.33	0.48	0.49	0.64
-18.12	**29_ts**	ΔE_R	0.02	0.07	0.10	-0.01	0.34	0.54	0.41	-0.09	-0.17	0.02	-0.06
10.87	**30_end**	ΔE#fwd	0.03	0.09	0.10	-0.03	-0.01	0.01	-0.07	0.04	0.16	0.06	0.18
19.87	**30_start**	ΔE#rev	-0.01	0.00	-0.01	-0.05	-0.12	0.10	-0.03	-0.01	0.04	-0.64	-0.58
-8.99	**30_ts**	ΔE_R	0.04	0.09	0.11	0.01	0.10	-0.10	-0.05	0.05	0.12	0.69	0.76
2.14	**31_end**	ΔE#fwd	0.03	0.09	0.07	-0.38	0.32	0.34	-0.36	0.28	0.49	2.36	2.56
12.37	**31_start**	ΔE#rev	0.00	0.05	0.05	-0.06	-0.09	0.20	0.98	0.20	0.22	2.18	2.20
-10.23	**31_ts**	ΔE_R	0.04	0.05	0.02	-0.32	0.41	0.14	-1.34	0.07	0.27	0.17	0.36
23.66	**32_end**	ΔE#fwd	-0.05	-0.08	-0.08	-0.09	-0.56	-0.83	-0.88	0.09	0.57	-0.02	0.44
58.44	**32_start**	ΔE#rev	0.04	0.18	0.18	0.10	0.35	0.34	0.72	0.01	0.54	0.09	0.61
-34.78	**32_ts**	ΔE_R	-0.09	-0.26	-0.26	-0.19	-0.91	-1.17	-1.61	0.08	0.03	-0.12	-0.17
2.77	**33_end**	ΔE#fwd	-0.02	-0.03	-0.03	-0.03	0.03	0.19	0.27	0.06	0.33	0.04	0.29
9.96	**33_start**	ΔE#rev	-0.03	-0.07	-0.07	-0.03	-0.07	0.19	-0.13	0.07	0.46	0.05	0.42
-7.20	**33_ts**	ΔE_R	0.01	0.04	0.04	0.00	0.10	0.00	0.40	-0.01	-0.13	-0.01	-0.13
28.85	**34_end**	ΔE#fwd	-0.03	-0.05	-0.03	-0.02	-0.21	0.07	0.67	0.20	0.23	1.08	1.11
4.32	**34_start**	ΔE#rev	-0.01	0.02	0.01	0.01	-0.05	0.23	-0.49	0.19	0.33	0.52	0.65
24.53	**34_ts**	ΔE_R	-0.02	-0.06	-0.04	-0.03	-0.16	-0.16	1.16	0.01	-0.10	0.56	0.45
14.97	**35_end**	ΔE#fwd	0.00	-0.01	0.00	0.17	0.63	1.02	2.02	0.16	0.38	0.40	0.61
-3.85	**35_start**	ΔE#rev	0.06	0.05	0.08	0.06	0.24	0.31	0.92	0.18	0.12	1.09	1.03
18.82	**35_ts**	ΔE_R	-0.06	-0.06	-0.08	0.11	0.39	0.72	1.10	-0.02	0.26	-0.69	-0.43

Table S2. Data for boxplot with quartiles and median from CCSD(T)/def2-SVP reference values (continued)

		LNO VeryTight	LNO Tight+wpt	LNO Tight	LNO Normal	DLPNO-T1 Tight	DLPNO-T0 Tight	DLPNO-T1 Normal	PNO Tight	PNO-T0 Tight	PNO Normal	PNO-T0 Normal
	MAX	0.57	1.44	1.55	2.42	11.09	12.63	15.02	3.27	3.25	7.10	7.14
	97.5%	0.26	0.47	0.47	1.07	3.50	3.14	4.07	2.92	2.83	4.35	4.25
	%90	0.07	0.18	0.19	0.32	0.82	1.18	1.73	0.35	0.81	1.21	1.58
box top	75%	0.02	0.09	0.08	0.13	0.40	0.58	0.87	0.19	0.39	0.50	0.66
median	50%	0.00	0.00	0.00	0.00	0.06	0.15	0.23	0.07	0.15	0.11	0.34
X	AVG	0.00	0.02	0.02	0.05	0.20	0.22	0.31	0.15	0.25	0.31	0.41
box bottom	25%	-0.05	-0.06	-0.07	-0.11	-0.17	-0.22	-0.38	-0.05	0.02	-0.20	-0.18
	10%	-0.09	-0.13	-0.14	-0.23	-0.78	-0.98	-1.32	-0.24	-0.29	-0.76	-0.89
	2.5%	-0.14	-0.26	-0.26	-0.37	-1.00	-2.01	-2.82	-0.61	-0.85	-1.45	-1.59
	MIN	-0.33	-0.94	-1.06	-2.23	-7.50	-8.55	-10.34	-2.01	-1.99	-4.30	-4.30
**RMSD**		**0.11**	**0.23**	**0.25**	**0.47**	**1.67**	**1.94**	**2.38**	**0.69**	**0.77**	**1.42**	**1.48**
**IQR (interquartile range)**		**0.07**	**0.15**	**0.15**	**0.24**	**0.57**	**0.80**	**1.26**	**0.23**	**0.37**	**0.71**	**0.85**
top whisker		0.13	0.31	0.31	0.50	1.26	1.78	2.76	0.54	0.94	1.57	1.94
bottom whisker		-0.16	-0.28	-0.29	-0.47	-1.03	-1.42	-2.27	-0.40	-0.53	-1.26	-1.45
top extreme fence		0.24	0.53	0.53	0.86	2.11	2.98	4.64	0.89	1.49	2.63	3.21
bottom extreme fence		-0.26	-0.50	-0.52	-0.84	-1.89	-2.61	-4.15	-0.75	-1.08	-2.33	-2.73

Table S3. Frozen core electrons per molecule in MOBH35 for all post Hartree-Fock calculations.

Molecule	Number of frozen core electrons
P_1	64
R_1	64
TS_1	64
P_2	56
R_2	56
TS_2	56
P_3	30
R_3	30
TS_3	30
P_4	28
R_4	28
TS_4	28
P_5	72
R_5	72
TS_5	72
P_6	58
R_6	58
TS_6	58
P_7	58
R_7	58
TS_7	58
P_8	76
R_8	76
TS_8	76
P_9	76
R_9	76
TS_9	76
P_{10}	40
R_{10}	44
TS_{10}	44
P_{11}	56
R_{11}	56
TS_{11}	56
P_{12}	66
R_{12}	66
TS_{12}	66

P_{13}	64
R_{13}	64
TS_{13}	64
P_{14}	42
R_{14}	42
TS_{14}	42
P_{15}	30
R_{15}	30
TS_{15}	30
P_{16}	20
R_{16}	20
TS_{16}	20
P_{21}	30
R_{21}	30
TS_{21}	30
P_{22}	42
R_{22}	42
TS_{22}	42
P_{23}	46
R_{23}	46
TS_{23}	46
P_{24}	92
R_{24}	92
TS_{24}	92
P_{25}	88
R_{25}	88
TS_{25}	88
P_{26}	36
R_{26}	36
TS_{26}	36
P_{27}	40
R_{27}	40
TS_{27}	40
P_{28}	60
R_{28}	60
TS_{28}	60
P_{29}	60
R_{29}	60
TS_{29}	60
P_{30}	40
R_{30}	40
TS_{30}	40

P_{31}	40
R_{31}	40
TS_{31}	40
P_{32}	30
R_{32}	30
TS_{32}	30
P_{33}	6
R_{33}	6
TS_{33}	6
P_{34}	34
R_{34}	34
TS_{34}	34
P_{35}	32
R_{35}	34
TS_{35}	34
H_2CO	4
CH_4	2
CO	4
PEt_3	22

R: reactant, P: product, TS: transition state.

Table S4. Total memory requirements (in GB) for local CC single point calculations for the product of reaction 16 of the MOBH35 dataset on two 8-core Intel Xeon E5-2630 v3 CPUs (2.40 GHz).

Methods	Threshold	def2-SV(P)	def2-SVP	def2-TZVP	def2-TZVPP	def2-QZVPP
DLPNO-CCSD(T)	NormalPNO	34.2	44.4	118.8	155.9	195.0
DLPNO-CCSD(T)	TightPNO (TcutPNO=10^{-6})	41.2	48.4	109.9	136.6	194.0
DLPNO-CCSD(T)	TightPNO (TcutPNO=10^{-7})	130.4	156.7	194.8	195.9	195.8
DLPNO-CCSD(T)	TightPNO (TcutPNO=10^{-8})	193.9	195.3	197.3	214.0	252.3
DLPNO-CCSD(T)	veryTightPNO	193.3	193.1	202.2	222.3	252.2
DLPNO-CCSD(T_1)	NormalPNO	34.5	44.7	118.8	156.6	196.7
DLPNO-CCSD(T_1)	TightPNO (TcutPNO=10^{-6})	41.6	48.8	110.6	137.2	202.1
DLPNO-CCSD(T_1)	TightPNO (TcutPNO=10^{-7})	130.4	156.9	197.8	197.2	196.6
DLPNO-CCSD(T_1)	TightPNO (TcutPNO=10^{-8})	193.9	195.3	197.0	213.9	252.3
DLPNO-CCSD(T_1)	veryTightPNO	193.3	193.9	201.7	222.9	252.3
PNO-LCCSD(T)	Default	60.0	60.1	60.4	77.3	232.7
PNO-LCCSD(T)	Tight	60.1	60.2	77.3	94.2	233.3
LNO-CCSD(T)	Normal	0.8	1.1	3.6	5.2	10.1
LNO-CCSD(T)	Tight	1.3	2.1	9.6	14.3	35.7
LNO-CCSD(T)	vTight	1.7	3.06	20.8	31.1	99.2
Canonical CCSD(T)		59.7	59.7	173.6	232.0	227.7
Nbasis		184	221	410	506	958

Table S5. Disk usage requirements (in GB) for local CC single point calculations for the product of reaction 16 of the MOBH35 dataset on two 8-core Intel Xeon E5-2630 v3 CPUs (2.40 GHz).

Methods	Threshold	def2-SV(P)	def2-SVP	def2-TZVP	def2-TZVPP	def2-QZVPP
DLPNO-CCSD(T)	NormalPNO	2.9	3.0	12.0	14.0	40.0
DLPNO-CCSD(T)	TightPNO (TcutPNO=10^{-6})	2.3	1.9	7.5	9.0	33.0
DLPNO-CCSD(T)	TightPNO (TcutPNO=10^{-7})	6.1	8.3	24.0	30.0	78.0
DLPNO-CCSD(T)	TightPNO (TcutPNO=10^{-8})	22.0	30.0	139.0	241.0	577.0
DLPNO-CCSD(T)	veryTightPNO	22.0	31.0	141.0	243.0	570.0
DLPNO-CCSD(T_1)	NormalPNO	15.0	23.0	70.0	78.0	139.0
DLPNO-CCSD(T_1)	TightPNO (TcutPNO=10^{-6})	18.0	21.0	50.0	68.0	107.0
DLPNO-CCSD(T_1)	TightPNO (TcutPNO=10^{-7})	29.0	46.0	122.0	149.0	287.0
DLPNO-CCSD(T_1)	TightPNO (TcutPNO=10^{-8})	57.0	75.0	139.0	242.0	727.0
DLPNO-CCSD(T_1)	veryTightPNO	57.0	75.0	243.0	324.0	728.0
PNO-LCCSD(T)	Default	10.1	13.4	43.8	54.9	127.7
PNO-LCCSD(T)	Tight	16.5	22.8	81.5	102.6	206.0
LNO-CCSD(T)	Normal	1.1	1.6	3.4	5.3	5.9
LNO-CCSD(T)	Tight	2.5	2.4	5.3	7.2	12.0
LNO-CCSD(T)	vTight	2.6	3.2	8.2	11.0	20.0
Canonical CCSD(T)		12.8	22.5	180.2	325.9	1300.0
Nbasis		184	221	410	506	958

Table S6. Correlation energy recovered from local coupled cluster methods and RMSD values (kcal/mol) for species in MOBH35 with respect to canonical CCSD(T)/def2-SVP.

		%E_{corr} recovered from CCSD(T)						%E_{corr} recovered from CCSD					
Methods	Cutoffs	avg.%[a]	avg.%[b]	avg.%[c]	RMSD[a]	RMSD[b]	RMSD[c]	avg.%[a]	avg.%[b]	avg.%[c]	RMSD[a]	RMSD[b]	RMSD[c]
DLPNO-CCSD(T)	Normal	99.10	99.13	99.14	2.55	1.46	1.39	99.73	99.75	99.76	1.24	0.80	0.71
DLPNO-CCSD(T_1)	Normal	99.42	99.44	99.46	2.38	1.30	1.17	99.73	99.75	99.76	1.24	0.80	0.71
DLPNO-CCSD(T)	Tight	99.41	99.42	99.44	1.95	0.99	0.90	99.71	99.72	99.73	1.11	0.57	0.50
DLPNO-CCSD(T_1)	Tight[d]	99.49	99.51	99.53	2.11	1.04	0.91	99.71	99.72	99.73	1.11	0.57	0.50
DLPNO-CCSD(T_1)	Tight[e]	99.77	99.79	99.80	1.67	0.78	0.56	99.87	99.88	99.89	0.88	0.46	0.34
DLPNO-CCSD(T_1)	Tight[f]	99.90	99.91	99.92	1.20	0.55	0.34	99.96	99.97	99.97	0.57	0.33	0.23
DLPNO-CCSD(T_1)	veryTight	99.89	99.90	99.91	1.14	0.50	0.29	99.94	99.94	99.95	0.55	0.28	0.19
Extrap{6,7}	Tight	99.98	99.99	100.00	1.39	0.70	0.40	99.99	100.00	100.01	0.76	0.45	0.32
Extrap{7,8}	Tight	100.01	100.02	100.03	0.82	0.42	0.27	100.04	100.04	100.04	0.37	0.30	0.26
PNO-LCCSD(T)	Default	99.67	99.68	99.69	1.44	1.11	0.78	99.79	99.80	99.81	0.90	0.76	0.57
PNO-LCCSD(T)	Tight	99.88	99.88	99.88	0.70	0.57	0.28	99.94	99.95	99.95	0.35	0.32	0.13
LNO-CCSD(T)	Normal	99.98	99.99	99.99	0.47	0.32	0.18	99.94	99.94	99.94	0.23	0.23	0.19
LNO-CCSD(T)	Tight	99.99	99.99	100.00	0.26	0.16	0.14	100.00	100.00	100.00	0.12	0.12	0.11
LNO-CCSD(T)	Tight+	100.00	100.00	100.00	0.25	0.16	0.15	100.01	100.01	100.01	0.11	0.11	0.11
LNO-CCSD(T)	vTight	100.00	100.00	100.00	0.14	0.12	0.11	100.02	100.02	100.02	0.08	0.08	0.08

[a] All reactions except for 17-20, 24-25; [b] Same but without reaction 9; [c] Same but without reactions 8 and 9; [d] TightPNO with TcutPNO = 10^{-6} E_h; [e] TightPNO with TcutPNO = 10^{-7} E_h, i.e. the Default TightPNO settings; [f] TightPNO with TcutPNO = 10^{-8} E_h.

Table S7. Correlation matrix of the MR character diagnostics.

	T1DIAG	D1DIAG	D2DIAG	FOD_TPSS	FOD_PBE0	%Ecorr[(T)]Kulik	max{t_i}^A	max{t_ij}^AB	|(T1)-(T0)|	(T1)/(T0)	1/H-L gap	Matito I_ND/I_tot	Matito revIND	Truhlar Mdiag	A100_TPSS	%TAE[ΔX]
T1DIAG	1.0000	0.9081	0.5194	0.4489	0.3422	0.4962	0.8288	0.0381	0.6466	0.5712	0.2756	0.2423	0.1928	0.1922	0.4838	0.2671
D1DIAG	0.9081	1.0000	0.7063	0.5512	0.5113	0.4816	0.8600	0.1534	0.7596	0.3671	0.4128	0.4328	0.2238	0.2864	0.2897	0.1797
D2DIAG	0.5194	0.7063	1.0000	0.6386	0.6577	0.5975	0.6208	0.4682	0.5706	0.0298	0.6596	0.5472	0.4294	0.5496	0.1985	0.2659
FOD_TPSS	0.4489	0.5512	0.6386	1.0000	0.8998	0.4682	0.4882	0.3533	0.4965	0.0848	0.7788	0.4617	0.3052	0.3521	0.0845	0.1155
FOD_PBE0	0.3422	0.5113	0.6577	0.8998	1.0000	0.4585	0.4473	0.3985	0.6234	-0.1437	0.8454	0.7063	0.3989	0.4636	-0.1339	-0.0185
%Ecorr[(T)]Kulik	0.4962	0.4816	0.5975	0.4682	0.4585	1.0000	0.4936	0.3118	0.5142	0.1786	0.4620	0.5139	0.8587	0.5618	0.5722	0.4654
max{t_i}^A	0.8288	0.8600	0.6208	0.4882	0.4473	0.4936	1.0000	0.1677	0.5540	0.4341	0.4576	0.2876	0.2171	0.3146	0.3212	0.2615
max{t_ij}^AB	0.0381	0.1534	0.4682	0.3533	0.3985	0.3118	0.1677	1.0000	0.2300	-0.1212	0.4327	0.3361	0.2827	0.3850	-0.0454	0.0720
|(T1)-(T0)|	0.6466	0.7596	0.5706	0.4965	0.6234	0.5142	0.5540	0.2300	1.0000	0.0933	0.3599	0.8392	0.3958	0.5711	0.1054	0.1089
(T1)/(T0)	0.5712	0.3671	0.0298	0.0848	-0.1437	0.1786	0.4341	-0.1212	0.0933	1.0000	-0.0265	-0.3406	-0.0992	0.0418	0.4945	0.2855
1/H-L gap	0.2756	0.4128	0.6596	0.7788	0.8454	0.4620	0.4576	0.4327	0.3599	-0.0265	1.0000	0.4470	0.3461	0.4418	-0.0862	-0.0158
Matito I_ND/I_tot	0.2423	0.4328	0.5472	0.4617	0.7063	0.5139	0.2876	0.3361	0.8392	-0.3406	0.4470	1.0000	0.5703	0.6799	-0.0596	0.0508
Matito revIND	0.1928	0.2238	0.4294	0.3052	0.3989	0.8587	0.2171	0.2827	0.3958	-0.0992	0.3461	0.5703	1.0000	0.5144	0.4135	0.2851
Truhlar Mdiag	0.1922	0.2864	0.5496	0.3521	0.4636	0.5618	0.3146	0.3850	0.5711	0.0418	0.4418	0.6799	0.5144	1.0000	0.0375	0.1919
A100_TPSS	0.4838	0.2897	0.1985	0.0845	-0.1339	0.5722	0.3212	-0.0454	0.1054	0.4945	-0.0862	-0.0596	0.4135	0.0375	1.0000	0.8134
%TAE[ΔX]	0.2671	0.1797	0.2659	0.1155	-0.0185	0.4654	0.2615	0.0720	0.1089	0.2855	-0.0158	0.0508	0.2851	0.1919	0.8134	1.0000

Table S8. Effects of core-valence correlation on forward and reverse barriers in LNO-CCSD(T) with Tight thresholds (kcal/mol)

	def2-TZVPP						cc-pwCVTZ(-PP)							
	$V_f^{‡}$, $\Delta E^{\#}_{fwd}$			$V_r^{‡}$, $\Delta E^{\#}_{rev}$			$V_f^{‡}$, $\Delta E^{\#}_{fwd}$			$V_r^{‡}$, $\Delta E^{\#}_{rev}$			$V_f^{‡}$	$V_r^{‡}$
**Rxn**	no (n-1)sp	with (n-1)sp	δ((n-1)sp)	no (n-1)sp	with (n-1)sp	δ((n-1)sp)	no (n-1)sp	with (n-1)sp	δ((n-1)sp)	no (n-1)sp	with (n-1)sp	δ((n-1)sp)	Δδ((n-1)sp)[a]	Δδ((n-1)sp)[a]
**1**	28.83	26.75	-2.07	15.72	13.90	-1.82	28.54	26.51	-2.03	15.80	14.68	-1.13	0.05	0.69
**2**	7.28	5.92	-1.36	24.05	22.29	-1.76	7.17	6.17	-1.00	23.89	22.22	-1.67	0.36	0.10
**3**	1.22	0.98	-0.24	26.19	26.05	-0.14	1.20	1.02	-0.18	25.90	25.83	-0.07	0.06	0.06
**4**	1.92	1.55	-0.38	7.93	7.76	-0.18	1.96	1.62	-0.34	7.90	7.70	-0.19	0.04	-0.02
**5**	4.92	4.69	-0.23	22.91	22.64	-0.27	4.71	4.47	-0.23	22.77	22.61	-0.16	0.00	0.10
**6**	15.46	15.47	0.01	14.71	14.59	-0.12	15.14	15.16	0.02	14.74	14.65	-0.09	0.01	0.03
**7**	27.57	27.58	0.01	18.88	18.85	-0.02	27.64	27.59	-0.05	18.95	18.96	0.01	-0.06	0.03
**8**	35.04	35.15	0.12	31.41	31.66	0.26	34.98	34.92	-0.06	31.76	31.81	0.05	-0.17	-0.20
**9**	27.72	28.32	0.59	12.78	11.59	-1.20	27.76	28.22	0.46	12.84	11.95	-0.89	-0.13	0.31
**10**	-2.99	-3.96	-0.97	11.05	9.28	-1.77	-3.41	-4.06	-0.65	10.32	9.47	-0.84	0.32	0.93

**11**	28.80	29.83	1.04	83.16	82.91	-0.25	28.25	28.89	0.64	82.99	82.63	-0.36	-0.40	-0.11
**12**	5.30	5.47	0.17	37.46	37.39	-0.07	5.32	5.39	0.07	37.49	37.41	-0.08	-0.09	-0.01
**13**	21.60	20.54	-1.07	49.30	48.58	-0.72	22.32	21.60	-0.72	48.85	48.36	-0.49	0.35	0.23
**14**	10.36	10.23	-0.14	14.24	14.39	0.15	9.98	9.86	-0.12	14.53	14.63	0.10	0.02	-0.06
**15**	20.17	20.83	0.65	73.82	74.72	0.89	21.37	21.94	0.57	73.78	74.33	0.55	-0.08	-0.34
**16**	34.37	35.50	1.12	54.28	53.60	-0.68	35.14	35.94	0.80	53.42	52.85	-0.57	-0.32	0.11
**21**	8.57	8.17	-0.40	8.57	8.17	-0.40	8.93	8.93	0.00	8.93	8.93	0.00	0.40	0.40
**22**	14.42	14.43	0.00	27.63	27.32	-0.31	14.34	14.27	-0.08	28.30	28.43	0.13	-0.08	0.44
**23**	30.22	29.96	-0.26	20.86	20.33	-0.53	30.51	30.13	-0.38	21.02	20.77	-0.25	-0.12	0.28
**26**	23.91	25.33	1.42	0.10	0.09	-0.01	25.74	26.25	0.50	0.03	0.04	0.01	-0.92	0.02
**27**	14.28	14.07	-0.22	1.85	1.92	0.08	14.24	14.24	0.00	2.29	2.32	0.03	0.22	-0.05
**28**	31.07	30.65	-0.42	15.95	15.87	-0.08	31.16	31.26	0.10	15.99	15.92	-0.07	0.52	0.02
**29**	15.29	15.27	-0.02	31.95	31.97	0.03	15.18	15.22	0.05	32.83	32.99	0.17	0.07	0.14
**30**	9.92	10.15	0.23	17.78	16.89	-0.88	9.80	9.91	0.11	16.74	16.19	-0.55	-0.12	0.34
**31**	4.51	3.28	-1.23	12.51	13.38	0.87	3.26	2.72	-0.55	13.36	13.73	0.37	0.69	-0.49
**32**	20.34	20.50	0.16	62.21	62.00	-0.20	20.53	20.53	-0.01	61.08	60.64	-0.44	-0.16	-0.23
**33**	1.06	1.20	0.15	9.27	8.11	-1.16	1.06	1.00	-0.07	8.66	8.18	-0.48	-0.22	0.68
**34**	28.02	29.74	1.72	4.14	3.44	-0.70	29.14	29.93	0.78	3.61	3.33	-0.28	-0.94	0.42
**35**	16.21	16.74	0.53	-2.16	-2.48	-0.32	16.69	17.13	0.44	-1.81	-1.74	0.08	-0.09	0.40

Table S9. Effects of core-valence correlation on forward and reverse barriers in LNO-CCSD(T) with Tight thresholds (kcal/mol)

	def2-{T,Q}ZVPP						cc-pwCV{T,Q}Z(-PP)							
	$V_f^{\ddagger}$, $\Delta E^{\#}_{fwd}$			$V_r^{\ddagger}$, $\Delta E^{\#}_{rev}$			$V_f^{\ddagger}$, $\Delta E^{\#}_{fwd}$			$V_r^{\ddagger}$, $\Delta E^{\#}_{rev}$			$V_f^{\ddagger}$	$V_r^{\ddagger}$
**Rxn**	no (n-1)sp	with (n-1)sp	δ((n-1)sp)	no (n-1)sp	with (n-1)sp	δ((n-1)sp)	no (n-1)sp	with (n-1)sp	δ((n-1)sp)	no (n-1)sp	with (n-1)sp	δ((n-1)sp)	Δδ((n-1)sp)[a]	Δδ((n-1)sp)[a]
**1**	28.69	25.72	-2.97	15.95	14.50	-1.46	28.80	26.41	-2.39	15.93	14.87	-1.06	0.58	0.40
**2**	7.51	5.71	-1.80	24.59	22.35	-2.24	7.49	6.35	-1.13	24.44	22.45	-1.99	0.67	0.25
**3**	1.22	0.92	-0.29	26.98	27.60	0.61	1.19	0.99	-0.20	27.00	26.44	-0.56	0.10	-1.17
**4**	2.17	1.38	-0.79	8.38	8.66	0.27	2.02	1.74	-0.28	8.36	7.87	-0.49	0.52	-0.76
**5**	5.53	4.90	-0.62	23.18	22.59	-0.58	5.56	5.33	-0.23	22.87	22.70	-0.17	0.39	0.41
**6**	15.75	15.88	0.12	14.95	14.83	-0.11	15.48	15.50	0.02	14.94	14.84	-0.10	-0.10	0.01
**7**	27.83	27.80	-0.03	18.78	18.71	-0.07	27.70	27.68	-0.02	18.73	18.61	-0.12	0.01	-0.05
**8**	34.96	33.51	-1.45	31.99	31.41	-0.58	35.06	N/A	N/A	32.11	32.22	0.11	N/A	0.70
**9**	29.56	29.56	-0.01	12.95	11.45	-1.50	29.45	29.69	0.25	12.54	11.50	-1.04	0.25	0.47
**10**	-1.97	-4.59	-2.62	9.23	7.77	-1.47	-2.08	-2.99	-0.91	9.53	8.93	-0.60	1.71	0.87
**11**	28.06	29.03	0.96	82.26	82.17	-0.09	27.96	28.58	0.62	82.23	82.27	0.04	-0.34	0.13
**12**	5.36	5.32	-0.04	37.42	37.19	-0.23	5.38	5.48	0.10	37.66	37.77	0.11	0.14	0.34
**13**	22.01	20.95	-1.06	49.04	48.32	-0.72	21.89	21.44	-0.45	49.45	49.02	-0.43	0.61	0.29
**14**	10.39	10.18	-0.21	14.70	14.86	0.16	10.16	10.06	-0.10	14.68	14.74	0.06	0.11	-0.10
**15**	19.62	20.21	0.58	75.44	76.07	0.63	19.97	20.20	0.23	75.55	76.02	0.47	-0.35	-0.16
**16**	34.58	35.34	0.76	54.79	54.25	-0.55	35.05	35.71	0.66	54.53	54.08	-0.45	-0.10	0.10
**21**	8.71	8.89	0.18	8.71	8.89	0.18	8.80	9.07	0.26	8.81	9.07	0.26	0.08	0.09
**22**	14.72	14.15	-0.56	27.81	28.03	0.22	14.60	14.47	-0.12	28.65	29.19	0.54	0.44	0.31
**23**	30.41	29.84	-0.57	21.16	20.89	-0.27	30.42	29.76	-0.66	21.24	21.10	-0.14	-0.09	0.13
**26**	24.74	25.54	0.80	0.13	0.17	0.04	26.06	26.31	0.25	0.16	0.14	-0.02	-0.55	-0.06
**27**	13.78	13.75	-0.02	2.31	2.47	0.16	13.51	13.45	-0.06	2.60	2.63	0.04	-0.04	-0.13
**28**	30.41	29.85	-0.55	15.69	15.55	-0.13	30.88	31.16	0.28	15.62	15.44	-0.18	0.83	-0.05

**29**	14.62	14.68	0.06	30.67	30.79	0.12	14.49	14.53	0.04	31.07	31.15	0.08	-0.02	-0.04
**30**	9.72	9.57	-0.16	17.68	16.97	-0.71	9.72	9.82	0.10	17.20	16.83	-0.38	0.26	0.33
**31**	4.21	2.85	-1.35	12.31	12.52	0.21	4.37	4.15	-0.23	12.63	12.76	0.13	1.13	-0.08
**32**	20.21	19.70	-0.51	64.13	64.18	0.05	20.06	20.02	-0.04	63.79	63.51	-0.28	0.47	-0.33
**33**	0.95	0.83	-0.12	8.95	8.04	-0.92	0.73	0.55	-0.18	8.91	8.72	-0.20	-0.06	0.72
**34**	27.72	28.27	0.55	3.56	2.84	-0.72	28.35	28.72	0.36	3.59	3.45	-0.14	-0.19	0.58
**35**	17.49	18.07	0.58	-2.09	-1.75	0.34	17.39	17.74	0.35	-1.96	-1.84	0.12	-0.24	-0.21
